# Quality of life, clinical characteristics and treatment adherence of people living with HIV/AIDS^1^

**DOI:** 10.1590/0104-1169.3534.2508

**Published:** 2014

**Authors:** Ana Cristina de Oliveira e Silva, Renata Karina Reis, Jordana Almeida Nogueira, Elucir Gir

**Affiliations:** 2 PhD, Adjunct Professor, Departamento de Enfermagem Clinica, Universidade Federal da Paraíba, Paraíba, PB, Brazil; 3 PhD, Professor, Escola de Enfermagem de Ribeirão Preto, Universidade de São Paulo, WHO Collaborating Centre for Nursing Research Development, Ribeirão Preto, SP, Brazil; 4 PhD, Full Professor, Escola de Enfermagem de Ribeirão Preto, Universidade de São Paulo, WHO Collaborating Centre for Nursing Research Development, Ribeirão Preto, SP, Brazil

**Keywords:** VHI, Acquired Immunodeficiency Syndrome, Quality of Life, Medication Adherence

## Abstract

**OBJECTIVES:**

to assess the quality of life of people living with HIV/AIDS and verify its association with clinical characteristics and treatment adherence.

**METHOD:**

cross-sectional study conducted in a hospital in the state of Paraíba, Brazil. A questionnaire was used to collect socio-demographic and clinical data. The quality of life scale proposed by the World Health Organization and a questionnaire to measure treatment adherence were used.

**RESULTS:**

of the 314 interviewees, 190 (60.5%) were male, aged 43 years on average, 121 (38.5%) had attended up to five years of schooling, 108 (34.4%) received up to two times the minimum wage, and 112 (35.7%) were on sick leave. In regard to clinical variables, individuals with an undetectable viral load scored higher in all the domains concerning quality of life, with statistically significant differences in three domains. Regarding treatment adherence, 235 (73.8%) presented poor adherence and those who strictly adhered to treatment obtained better scores in quality of life. The results show that quality of life is better among individuals adherent to ART. Supporting people to adhere to the antiretroviral treatment should be a persistent task of healthcare workers and other people participating in the treatment, such as family members and friends.

## Introduction

The impact of the antiretroviral therapy (ART) on people with HIV/AIDS contributed to a decrease in mortality or HIV/AIDS-related illnesses among infected people^(^ 1 ^)^. The number of AIDS-related deaths among adults and children decreased 29% since 2005. A decrease of 37% of AIDS-related deaths was observed in the last decade in Latin America^(^ 2 ^)^. This decrease in deaths is certainly a result of therapeutic support, which in addition to prolonging survival, also improves quality of life^(^ 3 ^)^.

Treatment adherence, psychological changes and clinical characteristics are factors directly associated with the quality of life (QoL) of people living with HIV/AIDS. Consequently, there is a search not only for preventive and control methods, but also for strategies that facilitate and improve the quality of life of HIV/AIDS patients. Coping with HIV/AIDS means facing situations that can decrease or limit quality of life^(^ 1 ^)^.

Health conditions, housing, safety and social relationships are factors that affect the quality of life of people living with HIV/AIDS in different respects. Quality of life is one of the terms with the highest multifactor expression, which justifies its many definitions. In this study, we opted for the definition of quality of life (QoL) proposed by the World Health Organization (WHO): "individuals' perceptions of their position in life in the context of the culture and value systems in which they live and in relation to their goals, expectations, standards and concerns"^(^ 3 ^-^ 4 ^)^.

In this context, the quality of life of people living with HIV/AIDS and its relationship with ART became an object of study. Many studies assess the QoL of these people, however, those addressing the relationship of QoL and ART are rare. Hence, this study's objective was to assess the quality of life of people living with HIV/AIDS and its association with clinical factors and adherence to antiretroviral therapy.

## Method

This analytical, cross-sectional study was conducted in a referral hospital for infectious diseases located in João Pessoa, Paraíba, Brazil.

This study included a convenience sample composed of 314 people living with HIV/AIDS. The participants were selected using the feasibility criterion on days care was provided in the referral hospital. The selection criteria included: living with HIV/AIDS, receiving ART for at least three months, and residing in the state of Paraíba, since many of this hospital's patients are from other states. Data were collected over a period of four months (December 2012 to March 2013) by properly-trained research assistants. The study was approved by the Institutional Review Board at the Federal University of Paraíba (Protocol No. 0405/12) and all the participants signed free and informed consent forms.

A semi-structured questionnaire was used to collect socio-demographic and clinical information. The WHOQOL-HIV Bref^(^ 5 ^)^ was used to assess Quality of Life. This instrument was composed of 31 items, divided into six domains: physical, psychological, social relationships, environments, level of independence, and religiosity^(^ 4 ^)^.

The CEAT-VIH instrument (*Cuestionario para la Evaluación de la Adhesión al Tratamiento Antirretroviral en Personas con Infección por VIH y Sida*) was used to collect data related to adherence to ART. This instrument is composed of 20 items divided into two dimensions: treatment adherence and factors influencing treatment adherence^(^ 6 ^)^. The minimum score is 17 and the maximum score is 89. In this study, the minimum score was 52 and the maximum was 88. In accordance with the analysis proposed by Remor^(^ 6 ^)^, the following percentages (PC) were established for the questionnaire global score obtained by the sample: PC ≥ 85 indicates strict adherence (good); 50 ≤ PC < 85 indicates poor adherence (difficulty complying with the treatment); PC ≤ 49 indicate no adherence. The scale's classification was used to compute the scores of each item of the WHOQOL HIV Bref. The internal consistency for the instrument domains was computed according to Cronbach's coefficient of reliability.

## Results

A total of 314 people living with HIV/AIDS participated in the study: 190 (60.5%) men and 124 (39.5%) women, aged 43 years old on average; ages ranged from 21 to 70 years old. Most participants (110/ 35.0%) were aged between 30 and 39 years old. In regard to education, 193 (61.4%) participants attended up to ten years of schooling, while individuals (121/ 38.5%) who attended from one to five years of schooling predominated. Most participants, 190 (60.5%), reported being Catholic; 190 (60.5%) individuals reported sick leave or retirement; and 226 (72%) individuals reported income of up to two times the minimum wage (Table 1).

**Table 1 -:** Distribution of people living with HIV/AIDS according to socio-demographic variables, João Pessoa-PB, Brazil, 2012 and 2013

Variables	Category	Individuals	
		No. of individuals (n)	Percentage (%)
Sex	Male	190	60.5
	Female	124	39.5
Age (years)	20 to 29	25	8.0
	30 to 39	110	35.0
	40 to 49	92	29.3
	50 to 59	64	20.4
	60 to 70	23	7.3
Race	Mixed	114	36.3
	Not mixed	200	63.7
Education (years of schooling)	None	27	8.6
	1 to 5 years	121	38.5
	6 to10 years	72	22.9
	11 to 15 years	74	23.6
	More than 15 years	20	6.4
Religion	Catholic	190	60.5
	Non-Catholic	102	32.5
	No religion	22	7.0
Income (minimum wage-MW)*	Less than 1 times the MW	118	37.6
	From 1 to 2 times the MW	108	34.4
	More than 2 times the MW	88	28.0
Occupation	Employed	74	23.6
	Retired	78	24.8
	Sick leave	112	35.7
	Unemployed	50	15.9
	Total	314	100

* Minimum wage current at the time of data collection was R$ 622.00/mo.

In regard to the distribution of individuals living with HIV/AIDS according to clinical variables (Table 2), sexual intercourse was the main route of exposure to the virus (251/79.9%). In regard to time since diagnosis, 124 (39.5%) individuals reported less than five years since diagnosis. In terms of clinical characteristics for the CD4 T cells count, most participants (220/70.1%) had more than 350 cells/mm^3^. The viral load was undetectable for 248 (79%) participants; from 50 to 100,000 copies per ml were observed for 59 (18.8%) individuals, and seven (2.2%) individuals presented more than 100,000 copies per ml (Table 2).

**Table 2 -:** Distribution of people living with HIV/AIDS according to clinical variables, João Pessoa-PB, Brazil, 2012 and 2013

Variables	Category	Individuals	
		No. of individuals (n)	Percentage (%)
Exposure to HIV	Sexual	251	79.9
	Vertical transmission	3	1.0
	Blood	6	1.8
	Others	54	17.2
Time since HIV diagnosis	0 to 5 years	124	39.5
	6 to 10 years	98	31.2
	11 to 15 years	78	24.8
	More than 15 years	14	4.5
Viral load (copies per ml)	< 50 (undetectable)	248	79.0
	50 to 100,000	59	18.8
	100,001 to 500,000	6	1.9
	> 500.001	1	0.3
CD4 T cells (amount of lymphocytes cells)	< 200 cells/mm^3^	30	9.6
	200 to 350 cells/mm^3^	64	20.4
	> 350 cells/mm^3^	220	70.1
	Total	314	100

In regard to time since diagnosis, Table 3 shows that all the individuals presented higher scores in the psychological and spiritual domains among those domains concerning quality of life, according to the WHOQOL HIV bref.

**Table 3 -:** Distribution of scores of the WHOQOL- HIV Bref domains according to clinical variables of people living with HIV/AIDS, João Pessoa-PB, Brazil, 2012 and 2013

Clinical variables		WHOQOL HIV Bref domains					
		Physical	Psychological	Level of independence	Social relationships	Environment	Spirituality /Religion
Time since diagnosis	3 months to 5 years	67.7 (18.9)	70.2 (13.1)	60.6 (15.0)	65.8 (17.5)	61.7 (15.1)	73.5 (21.2)
	6 to 10 years	67.9 (20.8)	69.1 (16.7)	60.7 (17.2)	66.2 (20.4)	60.6 (15.3)	69.6 (23.2)
	11 years or more	65.8 (20.9)	70.3 (16.6)	63.6 (14.8)	66.6 (18.9)	64.2 (14.7)	73.8 (21.5)
	P value	0.804	0.858	0.259	0.855	0.327	0.376
CD4 T (cell/mm^3^)*	< 200	69.8 (21.7)	67.0 (18.0)	56.5 (16.5)	66.3 (23.0)	57.8 (17.2)	76.0 (21.1)
	200 - 350	68.1 (20.0)	70.9 (15.2)	62.9 (17.6)	66.4 (17.9)	63.5 (13.0)	71.5 (23.2)
	> 350	66.6 (19.9)	70.0 (15.0)	61.8 (14.9)	66.1 (18.5)	62.3 (15.3)	72.2 (21.7)
	Valor de p	0.660	0.772	0.242	0.868	0.158	0.625
Viral load (copies per mL)^†^	<50 copies per ml	68.0 (20.1)	71.2 (15.0)	62.4 (15.5)	67.0 (18.4)	63.3 (15.3)	73.3 (21.5)
	≥50 copies per ml	64.4 (19.8)	64.9 (15.7)	58.0 (16.0)	62.8 (19.9)	57.7 (13.5)	68.9 (23.3)
	P value	0.114	0.004^‡^	0.033^‡^	0.113	0.009^‡^	0.163
Presence of another disease^ †^	No	67.2 (20.1)	70.0 (15.4)	61.5 (15.8)	66.1 (18.8)	62.0 (15.2)	72.4 (22.2)
	Yes	68.8 (19.8)	63.6 (10.3)	61.6 (9.8)	67.0 (19.0)	65.2 (10.4)	72.3 (9.4)
	P value	0.953	0.133	0.947	0.988	0.538	0.702
Previous diseases^†^	no	67.8 (19.9)	70.7 (14.8)	61.8 (15.7)	66.8 (18.8)	62.0 (15.3)	72.6 (22.2)
	yes	61.5 (21.4)	62.5 (18.2)	59.0 (14.8)	60.2 (18.0)	62.9 (13.6)	70.2 (19.7)
	P value	0.138	0.019^§^	0.236	0.097	0.896	0.405
Use of antiretroviral (months)^†^	Up to 60	68.3 (18.3)	71.0 (12.9)	60.4 (14.4)	66.6 (17.8)	61.5 (15.2)	72.9 (21.2)
	61 to 120	67.7 (22.9)	69.2 (17.7)	61.7 (17.3)	65.1 (19.9)	62.2 (15.7 )	71.5 (24.6)
	> 120	65.1 (19.6)	68.8 (16.1)	63.1 (15.6)	66.5 (19.2)	63.0 (14.4)	72.6 (20.3)
	P value	0.493	0.699	0.346	0.901	0.766	0.984

* Kruskal-Wallis test (comparison of three or more independent groups).

† Mann-Whitney test (comparison of two independent samples). Significant results: (‡) p-value< 0.01 and (§) p-value< 0.05

No significant differences were found in the WHOQOL HIV Bref regarding the counting of CD4 T lymphocytes. This study found significant differences in the association between viral load and the psychological, level of independence, and environment domains. Higher scores in all the QoL domains were identified among individuals with a viral load below 50 copies per ml.

As for the presence of diseases or comorbidities in association with QoL domains, no significant differences were found, though significant association was found in regard to the psychological domain and previous diseases. The clinical variable "use of antiretroviral" did not present significant difference in the QoL domains, according to the WHOQOL HIV Bref.

When the average scores of QoL were analyzed in regard to therapeutic adherence (Table 4), we verified that the individuals classified as "non-adherent" presented the worst scores in all the QoL domains. Table 4 presented statistically significant results for the domains: physical, psychological, social relationships, religion, and the assessment using the CEAT.

**Table 4 -:** Distribution of scores obtained in the WHOQOL- HIV Bref domains according to adherence measured by the CEAT of people living with HIV/AIDS. João Pessoa - PB. Brazil. 2012 and 2013

WHOQOL domains	CEAT ^+^ (adherence)	N valid	Average (scores)	Average rank	χ^2^ statistics	P value
Physical*	Strict adherence	44	70.5	172.1^†^		
	Poor adherence	235	68.2	161.6^†^	10.522	0.005^§^
	No adherence	35	56.4	111.9^‡^		
Psychological*	Strict adherence	44	73.5	183.8^†^		
	Poor adherence	235	71.3	164.0^†^	30.085	0.000^§^
	No adherence	35	55.7	81.0^‡^		
Level of independence	Strict adherence	44	61.2	156.7		
	Poor adherence	235	62.6	162.4	5.065	0.079
	No adherence	35	54.5	125.7		
Social relationships*	Strict adherence	44	72.6	183.2^†^		
	Poor adherence	235	66.2	157.6^‡^	8.158	0.017^||^
	No adherence	35	57.5	124.8^†^		
Environment	Strict adherence	44	64.1	174.2		
	Poor adherence	235	62.4	157.0	2.854	0.240
	No adherence	35	57.5	139.6		
Spirituality/ Religion*	Strict adherence	44	72.3	157.4^‡^		
	Poor adherence	235	73.9	163.0^‡^	6.799	0.033^||^
	No adherence	35	62.5	120.4^†^		

* Kruskal-Wallis test: significant results

† Dunn test (multiple comparisons): represent homogenous subsets ‡Dunn test (multiple comparisons): represent heterogeneous subsets

§ p-value < 0.05

## Discussion

The socio-demographic characterization found among this study's participants is in agreement with the profile of the HIV/AIDS epidemic in Brazil: men above older than 30 years old, with a low level of education and income, who were infected with HIV/AIDS through sexual transmission^(^ 2 ^)^.

Changes in the profile of people infected with HIV have drawn attention. The ratio of cases between men and women decreased in all age groups^(^ 7 ^)^. Even though the highest incidence of the disease is still observed among male individuals, recent studies show predominance among female individuals. Women are predominant in studies conducted in the South of India^(^ 8 ^)^ and in the United States^ (9)^.

Regarding clinical variables observed in this study, for time since diagnosis, the highest number of individuals (124/39.5%) was among those who became aware of their HIV-positive status less than five years ago. Most participants presented good clinical parameters concerning viral load, i.e., undetectable and with a high number of CD4 T cells. The individuals in this study with undetectable viral load scored higher in all the QoL domains. Increased viral load aggravates the individual's clinical condition, worsening symptoms and increasing the likelihood of hospitalization, consequently decreasing QoL^(^ 10 ^)^. Early diagnosis is one of the aspects linked to a favorable prognosis for HIV/AIDS. Being aware of one's serological status early on enables appropriate interventions and favors adherence to ART^ (11)^.

The results show that all the individuals scored well in the QoL domains according to clinical variables, especially in the psychological and spirituality domains, while the lowest scores were obtained in the level of independence and environment domains. The environment domain presented the lowest score in comparison to the other QoL domains in studies conducted in Brazil: Rondônia^(^ 12 ^)^, Santa Catarina^(^ 13 ^)^, in São Paulo^(^ 14 ^)^ and in Porto Alegre^(^ 5 ^)^.

One study developed in India reports that the scores obtained in QoL domains were higher in regard to the environment domain and lower in the domain concerning spirituality^(^ 8 ^)^. The inverse was observed in this study. Spirituality was the domain with the highest score. Religion, spirituality, and beliefs represent a search for meaning in life, a search for responses to why and for what purpose individuals face stressful and difficult situations, which are considered valid aspects for improved QoL^(^ 15 ^)^.

Spirituality provides support in times of distress, when there is social isolation and distance from the family. Religion is an important aspect providing meaning and order to life, suffering and distress, a result also found in another study^(^ 8 ^,^ 16 ^)^. An investigation conducted in Ribeirão Preto, SP, Brazil with individuals co-infected with tuberculosis and HIV/AIDS reports that the spirituality domains scored higher than the other domains^(^ 17 ^)^.

The total score obtained on the CEAT in this study was significantly correlated with the physical, psychological, social relationships, and spirituality/religion domains. Low scores obtained in the physical domain may be associated with clinical conditions, viral immunosuppression or lack of ART^(^ 17 ^)^.

Non-adherence to therapy, particularly among people living with HIV/AIDS, is a problem of greater magnitude. The consequences of patients' lack of adherence affect not only the non-adherent individual but also lead to a series of problems that may reach the population in general. Viral suppression, decreased contamination, minimization of opportunistic infections, and reduced resistance to antiretroviral agents depend on therapy adherence^(^ 18 ^)^.

Most of the studied sample present "poor adherence" and those classified as "non-adherent" obtained the lowest scores in all the QoL domains. The impact of non-adherence to ART on QoL was observed in this study. Given the changes in mortality and morbidity, studies show the positive impact of adherence to ART on QoL^(^ 18 ^)^.

It is known that adherence to ART improves clinical results, delays the progression of the disease, which in theory, should improve quality of life^(^ 16 ^)^. One longitudinal study reports that adherence to ART is associated with improved quality of life^(^ 19 ^)^. Cross-sectional studies also identified associations between QoL and adherence to ART^ (10,20)^.

When assessing the quality of life of individuals with HIV/AIDS and its association with ART, one needs to identify the individual's clinical characteristics and which phase of the infection the individual is experiencing: the asymptomatic, symptomatic or HIV/AIDS last stage^(^ 21 ^)^.

 Adherence to antiretroviral treatment may cause side effects in the short run (nausea and vomiting) and in the long run (lipodystrophy and dyslipidemia), impacting quality of life. One study reports no significant difference in the domains of QoL in association with the use of antiretroviral agents, but found significant difference in the overall assessment of health with the presence of adverse effects of medications^(^ 21 ^)^.

The relationship between adherence to ART and quality of life is reciprocal. The quality of life of people living with HIV/AIDS may be influenced by adherence to ART. In this context, it is imperative to understand antiretroviral therapy in order to maximize its benefits and minimize its adverse effects^ (21)^.

Most of the participants in this study were asymptomatic with undetectable viral load (< 50 copies per ml). It can be inferred that these clinical characteristics influence adherence to ART and, consequently, influence the QoL of individuals because the absence of symptoms combined with the presence of undesirable side effects, as reported by another study, are factors that hinder adherence to therapy^(^ 22 ^)^.

One limitation of this study is the cross-sectional methodological design. Despite this limitation, the results indicate the need to assess the influence of ART on QoL in this specific population. 

## Conclusion

The quality of life of the studied population according to the WHOQOL HIV Bref was associated with clinical variables and adherence to antiretroviral therapy. Regarding the clinical variables, it is important to note that individuals with viral load below 50 copies per ml obtained the highest scores in all the QoL domains. As for adherence to antiretroviral therapy, individuals classified as "non-adherent" obtained the lowest scores in all the QoL domains.

Adherence to antiretroviral therapy is a positive factor in quality of life, especially because it improves immunity, controls viral load and delays the progression of the disease. Despite these benefits, many people living with HIV/AIDS have difficulty adhering to the therapy. Such difficulties are not only related to the clinical repercussions of the treatment but also due to difficulties accessing the service, and consequently, accessing medications. 

In this sense, we suggest there is a need to monitor the ART adherence of people living with HIV/AIDS based on the understanding that adherence is a continuous process that mainly involves the family and healthcare workers. In some situations, non-adherence results from the family and the healthcare workers themselves abandoning the patient.
